# POInT_browse_: orthology prediction and synteny exploration for paleopolyploid genomes

**DOI:** 10.1186/s12859-023-05298-w

**Published:** 2023-04-27

**Authors:** Mustafa Siddiqui, Gavin C. Conant

**Affiliations:** 1 Department of Biological Sciences, North Carolina State University, Raleigh, NC USA; 2 Bioinformatics Research Center, North Carolina State University, Raleigh, NC USA; 3 Program in Genetics, North Carolina State University, Raleigh, NC USA

**Keywords:** POInT, Comparative genomics, Browser

## Abstract

We describe POInT_browse_, a web portal that gives access to the orthology inferences made for polyploid genomes with POInT, the Polyploidy Orthology Inference Tool. Ancient, or paleo-, polyploidy events are widely distributed across the eukaryotic phylogeny, and the combination of duplicated and lost duplicated genes that these polyploidies produce can confound the identification of orthologous genes between genomes. POInT uses conserved synteny and phylogenetic models to infer orthologous genes between genomes with a shared polyploidy. It also gives confidence estimates for those orthology inferences. POInT_browse_ gives both graphical and query-based access to these inferences from 12 different polyploidy events, allowing users to visualize genomic regions produced by polyploidies and perform batch queries for each polyploidy event, downloading genes trees and coding sequences for orthologous genes meeting user-specified criteria. POInT_browse_ and the associated data are online at https://wgd.statgen.ncsu.edu.

## Background

**Fig. 1:**
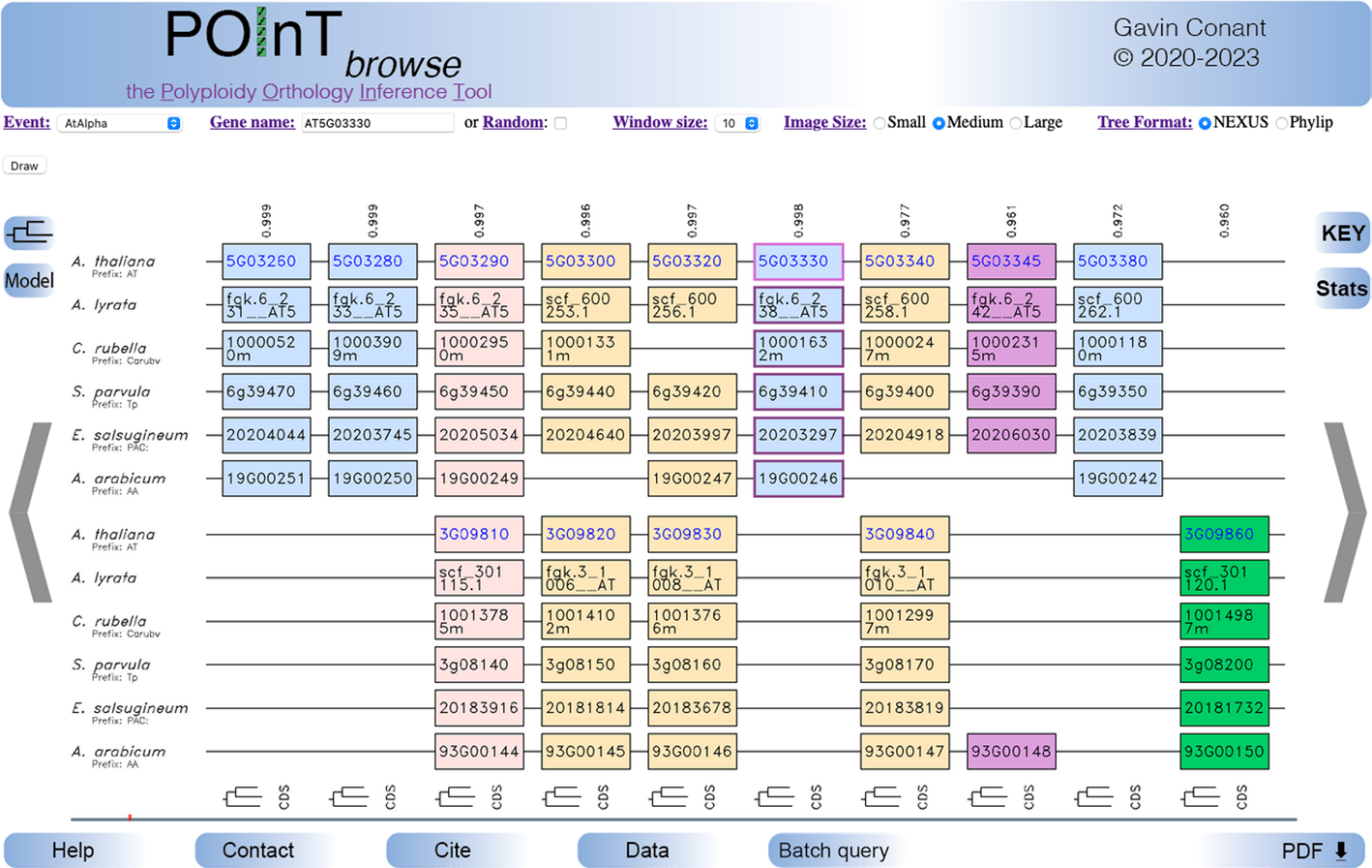
An example visualization from POInT_browse_. Shown is a region of ten duplicated regions (“pillars”) with between 6 and 12 surviving genes from six species sharing the At-α polyploidy event. The view is centered on the selected gene from *Arabidopsis thaliana,* AT5G03330 (pink outline). Users control the visualization size, window size and the format of the downloaded gene trees from the top controls. The tree pictogram on the upper left gives the assumed species phylogeny, while the “Model” button gives a visualization of the duplicate loss model used for the event (including model parameters). At right, the “Key” button illustrates the color scheme while the “Stats” button gives the POInT_browse_ version, the size of the current dataset and the sizes of all datasets currently in POInT_browse_. Users can navigate ½ frame left or right with the arrows or re-center the frame on a pillar by clicking on it. Hovering over a gene gives its chromosomal coordinates and its common name (if known). Gene names shown in blue link to the corresponding model organism gene database entry for that gene. The location of the current frame relative to the full set of pillars is shown with the red region in the blue-gray bar at the bottom. This bar can also be used for coarse navigation within the pillars of an event. The upper panel with some blue genes shows the less fractionated subgenome, the bottom, the more fractionated one (green). Light pink genes are fully retained as duplicates and the darker pink pillar illustrates a reciprocal gene loss. Pillars with a mix of duplicated and single-copy genes are shown in tan. Numbers at the top of each pillar are POInT’s confidence estimate (0.1) for the orthology relationships shown (see text). At the bottom of each pillar, the tree pictogram will download a gene tree with the corresponding orthology relationships for the genes in that pillar; the “CDS” button will download the coding sequences of the genes in question. A PDF version of the current window can be downloaded from the “PDF” button at right; the “Batch query” button opens a new window with the batch download interface

Ancient polyploidy events are widely distributed across the eukaryotic tree [1]. At the time of their formation, polyploid organisms have four (or more) complete sets of chromosomes in their nucleus [2], which can be thought of as a duplication of every gene in the genome (hence whole-genome duplication or WGD). This fully duplicated state is transitory and followed by the rapid loss of many of these duplicated genes [3]. Such losses may occasionally be due to selection [4] but probably most commonly occur through neutral processes [5, 6]. The losses can also occur both prior to or after speciation events among the taxa sharing the polyploidy. Losses result in a distinct pattern of double-conserved synteny (DCS) between the surviving genomes (Fig. 1), where the pre-polyploidy genome order can be reconstructed by merging the two duplicated regions, each of which preserves a fraction of the original gene content. Many of these events are *allopolyploidies*, meaning that the genomes that merged were not identical, making the event a combination of a hybridization and a genome doubling. For such events, it is common to observe that one of the progenitor genomes is favored among the surviving single-copy genes, a pattern known as *biased fractionation* [7]. This pattern is illustrated in Fig. 1: the excess of blue columns relative to green ones is the result of duplicate losses more commonly coming from the lower subgenome than from the upper one.

**Fig. 2:**
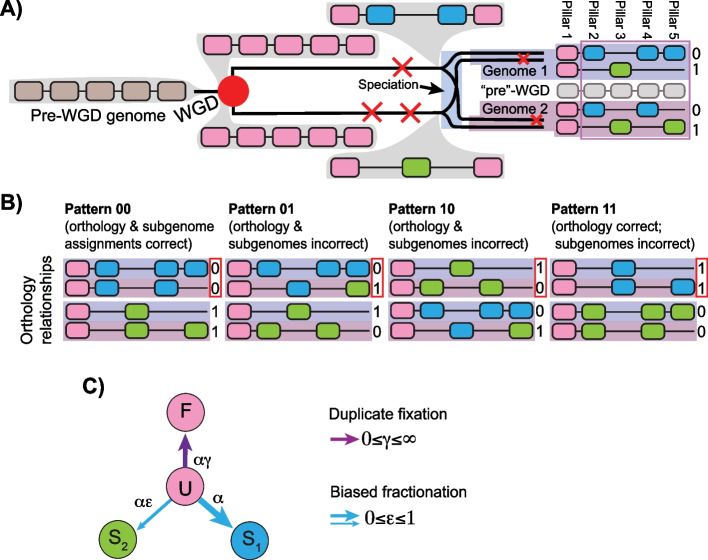
Polyploidy, genome evolution and the POInT computation. **A** A polyploidy event followed by a speciation and duplicate gene losses produces regions of double-conserved synteny (DCS) in the two resulting genomes, raising the issue of phasing those regions with respect to each other. A region of five genes (brown) in the non-polyploid ancestor is duplicated (pink) and experiences three duplicate gene losses prior to a speciation event (red “X”s). After the speciation event, the two resulting species also experience independent losses, yielding the blocks of DCS in each genome. **B** There are 2^*n*^ = 4 possible orthology relationships for the DCS blocks in these *n* = 2 genomes. These relationships are shown for the example (known) loss patterns from **A**. **C** For each orthology relationship in **B**, the likelihood of the observed presence/absence data at that pillar can be computed on the species phylogeny with a Markov model of duplicate losses. Those likelihoods can then be conditioned on the other pillars in the dataset. In this model, undifferentiated duplicate genes (*U*) can either be fixed (*F*) or lost from subgenome 2 (copy 1 or *S*_1_ survives) or lost from subgenome 1 (*S*_2_ survives). As the ε parameter (0 ≤ ε ≤ 1) approaches 0, subgenome 1 is increasingly favored over subgenome 2. Model parameters and tree branch lengths are estimated from the pillar data by maximum likelihood [9]. From this model, the relative likelihood of each of the orthology relationships in **B**, conditional on the full dataset, can be computed: these values are the confidence estimates at the top of the pillars in Fig. 1

Both duplicate losses and biased fractionation introduce complications for comparative genomics. Although DCS patterns are evident in any polyploid genome, it can be difficult to determine which region of any such genome is orthologous to a given region in a related genome [8]. For a genome duplication (tetraploidy) shared by *n* genomes, there are 2^*n*^ possible orthology relationships at each locus (“pillar” in Fig. 1). As shown in Fig. 2, the potential for independent duplicate gene losses in different genomes sharing a polyploid event can make identifying the “true” orthology relationship difficult. This difficulty can confound functional analyses, phylogenetics and studies in molecular evolution.

To address this problem, we developed POInT (the Polyploidy Orthology Inference Tool), a phylogenetic modeling approach to studying shared polyploidies [8]. POInT uses a hidden Markov model to combine a phylogenetic model of duplicate loss after polyploidy with synteny information to infer which of these 2^*n*^ possible orthology relationships is most likely. The POInT computation has been described several times [8, 10, 11]. In Fig. 2, we give a cartoon overview. The polyploidy event leaves DCS as its hallmark. Duplicate gene losses leave “holes” in the DCS blocks that may be common to all species with the event or restricted to some clades (Fig. 2A). Since for real genomes we cannot know the true history (as we do for Fig. 2A), we employ a user-specified model of duplicate gene loss (Fig. 2C) to compute the likelihood of every possible orthology relationship (Fig. 2B) at every pillar, conditioned on all possible relationships at every other pillar and their syntenic relationships. At each pillar, the confidence in the inferred orthology relationship in Fig. 1 is then simply the likelihood of that orthology relationship at that pillar, conditional on every other pillar, over the total likelihood of the dataset. These confidence values are noted at the top of each pillar in Fig. 1.

Further development of POInT allowed us to model genome triplications (hexaploidy events) and biased fractionation [11, 12]. POInT now provides a statistical framework for testing hypotheses such as the presence and strength of biased fractionation and whether pairs of single-copy genes in different genomes are orthologs or are paralogs created by losses of alternative copies of the duplicate pair. Here we describe the POInT_browse_ portal (wgd.statgen.ncsu.edu), which gives access to all of these data both for browsing and for download.

## Construction and content

POInT is written in c++ with dependencies on the LAPACK linear algebra libraries [13] and the GNU plotutils package; it is parallelized with OpenMP [14]. POInT_browse_ is a c++ CGI front-end that communicates with daemonized copies of POInT through UNIX interprocess communication. Hence, each running copy of POInT stores the computed orthology inferences for particular polyploidy event. When the CGI frontend sends a request for a browser frame from a given event, the appropriate POInT instance determines the best orthology relationship for each pillar in the requested window. It then creates the visualization in PNG format and returns that image to the browser. The generation of gene trees is handled in a similar manner.

**Fig. 3:**
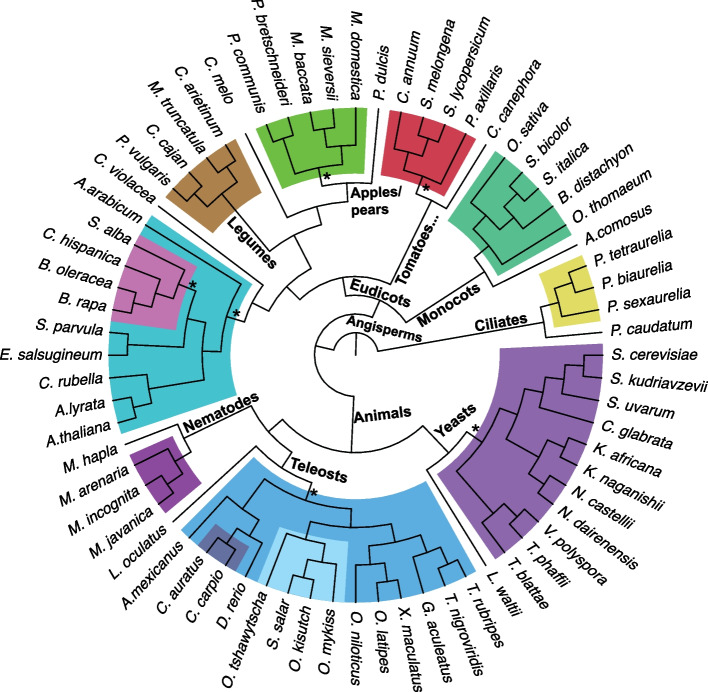
Evolutionary relationships of the twelve polyploidy events in POInT_browse_. The twelve polyploidy events currently displayed in POInT_browse_ are shown as colored blocks with the assumed (labeled by “*”) or computed (all others) species trees used by POInT depicted. We used a non-polyploid outgroup genome to infer the DCS blocks for each event, and these organisms are shown uncolored. References to our analyses of these events are given in the text and at the POInT_browse_ website

To date, we have used POInT to analyze twelve polyploidy events, comprising 59 genomes and > 600,000 coding genes (Fig. 3), all available from POInT_browse_. Of these twelve events, analyses of ten have been previously published, including the yeast WGD [15], the At-α event in *A. thaliana* and its relatives and the grass ρ event [11], the teleost genome duplication [16], hexaploidies in Brassiceae [12] and Solanaceae [17], a triploidy in parasitic nematodes [18] and WGD events in salmonids, paramecia and legumes [5]. The POInT_browse_ documentation gives accession numbers and genome publication references for all twelve events.

POInT_browse_ has three core functions. First, users can enter a gene identifier from one of the polyploid genomes and generate a visualization of the genomic region around that gene, including the corresponding orthologous and paralogous region(s) in the other polyploid genomes modeled (Fig. 1). Users can then step through the inferred regions with the provided arrows or recenter the view on a particular pillar by clicking on it. These interface details are borrowed from the Yeast Gene Order Browser [YGOB; 18]. The track at the very bottom of Fig. 1 illustrates the location of the current window relative to the entire set of pillars: clicking on this track allows the user to make larger jumps through the pillars. Any visualization generated can be downloaded as an Adobe PDF file for presentation.

POInT_browse_’s second function is to allow users to download predicted gene trees and/or coding sequences for any selected pillar in a browser frame by clicking on the icons at the bottom of each pillar (Fig. 1). These gene trees are created by combining the assumed species tree for that polyploidy event (available from the button on the upper left) with POInT’s orthology inferences. For example, in the case of a fully duplicated column, the gene tree returned will consist of two mirrored copies of the species tree with the gene identifiers from the orthology predictions at the tips. In cases where duplicate losses have occurred, those tips are pruned from the species tree.

POInT_browse_’s final capacity is a batch download feature, reached with the “Batch query” button (Fig. 1). This button opens a new window where the user selects a polyploidy event from which to download orthology inference sets. Pillars from that event can be selected based on orthology confidence combined with specifications for the number of duplicate genes required to be present (from fully duplicated to fully single-copy). Alternatively, the query can be restricted to single-copy orthologs. In each case, POInT returns a UNIX tar file containing CDS regions and gene trees meeting the selected criteria. Thus, when single-copy orthologs are requested, the download includes pillars where only a single gene survives from the polyploidy event in each genome and where POInT predicts all of these genes to be orthologs at the confidence level selected. In this case, the user can also request only orthologs from the less or more fractionated genome, again based on POInT’s inferences.

## Utility and discussion

POInT and POInT_browse_ represent an advance on other polyploid-genome visualization tools [19, 20] for several reasons: in particular they allow hypothesis testing through differing models of duplicate loss [8, 16] and provide confidence estimates for their orthology inferences. Of course, as with any approach, there are limitations to the POInT framework. POInT assumes that duplicate losses are independent along a chromosome and follow an assumed species phylogeny, both of which may be violated in some cases [21]. Even if we accept POInT’s modeling framework, datasets where the genomes considered are highly fragmented can result in generally low confidence in the orthology inferences, as is seen for the triploid nematodes [18].

Given these advantages and disadvantages, how can POInT_browse_ help researchers? It is targeted to three groups: those studying processes associated with polyploidy, such as biased fractionation, those interested in phylogenomic questions, and users interested in molecular evolution more generally. The value of synteny-based orthology data is illustrated in each case by prior work using either data from POInT or from YGOB [19], which was the antecedent to POInT. As an example of the first case, namely the study of polyploidy, we used the synonymous divergence of conserved duplicates to assess the relative rate of duplicate loss immediately after polyploidy relative to the loss rate later in the history of those lineages. We found that many, but not all, polyploidy events were characterized by an especially rapid loss of duplicated genes immediately after the event [5]. Likewise, Marcet-Houben and Gabaldon [22] used data from YGOB, among other sources, to present phylogenetic evidence that the yeast genome duplication was an allopolyploidy. We have also used the inferred orthologs from POInT to test whether repetitive element distributions differed between the subgenomes of extant mesohexaploid vegetable crops [17].

In the case of phylogenetics, polyploidy causes at least two difficulties in tree inference. The mere presence of duplicated genes makes the problem of reducing gene trees to species trees complex [23]: the common solution to this problem is to use only single-copy genes in large-scale analyses [24]. However, even in this framework, the loss of duplicated genes, and in particular, the *reciprocal* loss of duplicated genes in different taxa (dark pink column in Fig. 1), can give rise to cases where single-copy genes in multiple genomes are not orthologous [3]. The rate of reciprocal gene loss varies considerably across polyploidy events but is a universal feature of post-polyploid evolution [5]. Since reciprocal gene loss has been shown to adversely affect the quality of phylogenies inferred for polyploid taxa [25], using synteny information to restrict analyses to true orthologs is a promising approach for phylogenetic analyses of paleopolyploid taxa [26]. POInT_browse_ potentially provides a route around both of these problems, giving researchers access to any desired set of orthologous genes, single-copy or otherwise, from which to start the inference process.

The final utility of POInT_browse_ is for more general questions regarding the molecular evolution of duplicated genes. Deluna et al., [27] have used YGOB data to explore how duplicated genes do or do not contribute to robustness to gene loss, while Gera et al., [28] used WGD-produced duplicated transcription factors (identified with YGOB) to explore the post-WGD divergence in their binding specificity. Understanding the paralogous structure of a genome using tools like YGOB has also been critical for detecting neofunctionalization: the appearance of novel functions through gene duplication [29]. Finally, we have used orthology data from POInT to study post-polyploidy gene conversion [30–32]. Because POInT provides high quality orthology inferences that are not dependent on gene trees inferred from the sequences involved, the orthology that is evident from the gene order can be contrasted with gene trees inferred from the sequences. In our case, we could show that paralogous ribosomal proteins showed evidence for very strong and recent gene conversion, such that those paralogs, created by the ancient genome duplication about one hundred million years ago [33], were more similar to each other than either was to its orthologous gene in a closely related yeast species, despite the much more recent split (a few million years) of those orthologs [31].

## Conclusions

POInT_browse_ is a freely available collection of orthology inferences for more than fifty polyploid genomes from across the eukaryotic tree of life. The syntenic regions, gene sequences and inferred gene trees can be useful for researchers studying polyploid genome evolution, systematics and molecular evolution more generally.

## Data Availability

*Project name:* POInT_browse_. *Project home page:* wgd.statgen.ncsu.edu. *Operating system:* Platform independent. *Programming language:* c++. *Other requirements:* Web browser. *License:* LGPL-3.0. *Other restrictions:* None. POInT_browse_ is available at wgd.statgen.ncsu.edu; the full POInT software package (v1.61), including the browser code, is available at https://github.com/gconant0/POInT. All of the data distributed through POInT_browse_ are also available for download directly from the POInT_browse_ website.

## Abbreviations

WGD: Whole-genome duplication
DCS: Double-conserved synteny
